# Temperament and Hunger Interact to Determine the Emergence of Leaders in Pairs of Foraging Fish

**DOI:** 10.1371/journal.pone.0043747

**Published:** 2012-08-29

**Authors:** Shinnosuke Nakayama, Rufus A. Johnstone, Andrea Manica

**Affiliations:** Department of Zoology, University of Cambridge, Cambridge, United Kingdom; CNRS, Université de Bourgogne, France

## Abstract

Studies on leadership have focused either on physiological state as the key predictor (i.e. “leading according to need”), or else on temperamental asymmetries among group members (i.e. intrinsic leadership). In this paper, we explore how both factors interact in determining the emergence of leaders. We observed pairs of sticklebacks with varying degrees of temperamental difference, and recorded their movements back and forth between a safe covered area and a risky foraging area, both before and after satiating one of the two pair members (but not the other). Before satiation, when the fish had similar hunger levels, temperament was a good predictor of social roles, with the bolder member of a pair leading and the shyer member following. The effect of satiation depended on which fish received the additional food. When the shyer member of a pair was fed, and consequently became less active, the bolder fish did not change its behaviour but continued to lead. By contrast, when the bolder member of a pair was fed, and consequently initiated fewer trips out of cover, the shyer partner compensated by initiating trips more frequently itself. In pairs that differed only a little in temperament, feeding the bolder fish actually led to a role reversal, with the shyer fish emerging as a leader in the majority of joint trips out of cover. Our results show that leadership emerges as the consequence of multiple factors, and that their interaction can be complex.

## Introduction

To work effectively as a group, animals must coordinate their activities [1]. However, unless all group members have identical preferences, there will be a price of consensus, as some individuals have to make compromises on their preferred timing or course of movement in order to ensure group cohesion [2]. In a democratic society, the cost of consensus would be fairly spread among all group members [3]. In reality, there is a growing body of evidence that some individuals (‘leaders’) are able to influence group behaviour disproportionately, with other group members (‘followers’) accepting their lead [4]–[13]. A key question in social biology is to determine which factors predict the tendency of an individual to emerge as a leader.

Why do some individuals end up imposing their will on the rest of the group? One possible explanation is that they stand to gain the most if the group adopts their preferences. For example, female zebras have been shown to initiate and lead movement of a herd more often during lactation, when their energetic needs are most demanding [7]. Such leadership “according to need” has received a good deal of empirical support [5], [8], [14]–[16], and has been the focus of most theoretical models investigating the evolution of leadership [17]–[20]. Rands et al. [17] showed that, even in the absence of underlying asymmetries among group members, small differences in need that arise merely through chance variation in foraging success can be perpetuated during social foraging. Hungry individuals rarely catch up other group members who have larger energy reserves, thus forcing them to keep leading new trips to look for more food; as a result, individuals become locked into leader and follower roles.

A different explanation for the emergence of leaders and followers in animal groups is that some individuals are more influential and/or less responsive to others. Individual temperament has received a good deal of attention over the last few years as an explanation of behavioural variation within a group [9], [21]–[25]. For example, in pairs of foraging sticklebacks, bolder individuals tend to lead trips out of cover to a potential food source [9], [13]. In this case, leadership arises from the greater willingness of certain individuals to take the risk of being the first to leave cover, something that shy individuals are reluctant to do. Recent theoretical work has shown that individual variation in leadership ability can be expected even in the absence of any competitive asymmetry among group members, simply as a consequence of the need to coordinate and avoid costly disagreements [25].

These two explanations of leadership are not mutually exclusive, even though many discussions of animal temperament draw a contrast between the idea of consistent individual differences in behaviour, on the one hand, and the possibility of flexible behavioural tactics based on current state, on the other (reviewed by [26]). We do not know of any study that tests the interplay of physiological state and temperamental differences. To investigate the interaction between these two factors, we therefore set up pairs of sticklebacks with different degrees of temperamental differences, and observed their foraging behaviour both when they had similar hunger levels and after one of the pair members (but not the other) had been satiated.

## Materials and Methods

### Animal Collection and Maintenance

About 200 three-spined sticklebacks were collected, in August 2010, from a small branch of the River Cam, about 10 miles northeast of Cambridge (England, UK), using a sweep net. Fish were immediately brought back to our laboratory at the University of Cambridge, and kept in a large glass holding aquarium (120×60×60 cm) with artificial plants, aeration and an under-gravel filtration system. Fish were fed frozen bloodworm (Chironomidae) larvae *ad libitum* once daily. Temperature was kept at 16±1°C, and photoperiod was set at 10-h light 14-h dark cycles. The sex of the fish was not identified, as the temperature and photoperiod regime prevented them from becoming sexually mature [27]. Fish were allowed to acclimate in the holding aquarium for at least one month before being used in any experiment. Animal care and experimental procedures were approved by the Animal Users Management Committee of the University of Cambridge under a non-regulated procedures regime.

### Temperament Assessment

Before starting the main experiment, we estimated individual temperament (measured as the proportion of time an individual spent out of cover in a standardised setup while in isolation). To allow identification, 60 fish of similar sizes (35–42 mm in standard length, mean 37.8 mm) were housed in individual compartments in custom holding tanks (60×30×40 cm, each divided into six compartments by transparent dividers). Each compartment contained an artificial plant at one end and, at the other end, a white plastic plate (2×2 cm) where food was delivered. Fish were fed bloodworms when they missed feeding during the training sessions described below. Under-gravel filtration systems were used to maintain water quality.

All fish went through an initial training period during which they learned to expect food at a specific feeding area in the experimental tank. The experimental tank (90×30×30 cm) was divided lengthwise with an opaque, white plastic partition to create two long lanes. Each lane was lined with gravel, which was sloped to create a deep (12 cm depth) ‘safe’ area, in which an artificial plant was placed to provide cover, and a shallow (2 cm depth) ‘risky’ area, in which food was delivered on a white feeding tile (2×2 cm). A white plastic tile (8×4 cm) was placed vertically in front of the feeding tile to prevent fish from seeing whether food was present while staying at the deep end. At the start of each training session, an individual fish was transferred at the deep end using a dip net, and a single medium-sized bloodworm was placed on the feeding plate. An observer checked the feeding tile after 30 min, and added a second bloodworm if the first had been consumed. One hour after the start of the training session, food was checked again, and fish were put back into their individual compartments. Fish that failed to consume two bloodworms during the training session were given the appropriate amount of food in their holding compartment to maintain similar energy reserves across all individuals. Each fish went through three one-hour training sessions over consecutive days; fish that failed to consume any food during the final training session were excluded from the experiments.

The day after the last training session, fish temperament was assessed in a one-hour session in the experimental tank during which no food was presented (to avoid eliciting localised search behaviour in the risky area once a food item was found). Fish behaviour during the temperament assessment was recorded from above using a camcorder (Toshiba Camileo ×100, Toshiba Corporation, Japan). The timing of leaving and returning to cover was subsequently recorded from the video playback using a custom-designed data logger. An individual boldness score was then estimated as the proportion of time spent out of cover over the observation period. Among 120 individuals tested, boldness scores ranged from 0.02 to 0.81 (mean 0.31, median 0.30, SE 0.02). Individual boldness of this species is known to be highly repeatable [9], and we have used this measure of boldness in a number of experiments [9], [13], [28]–[30].

### Experimental Procedure

We wanted to test whether feeding one member of a pair affected collective behaviour differently depending on the temperament of the fed fish relative to that of its partner. To answer this questions, we set up four categories of pairing, in which pair members differed in temperament in four possible ways: the focal fish (which eventually was fed) was moderately shyer than its partner (coded as focal Shy Moderate difference, SM; focal fish's boldness score 70–85% of the partner's score, *n* = 14), the focal fish was a lot shyer than its partner (focal Shy Large difference, SL; <50%, *n* = 11), the focal fish was moderately bolder than its partner (focal Bold Moderate difference, BM; 115–140%, *n* = 25), and the focal fish was a lot bolder than its partner (focal Bold Large difference, BL; >200%, *n* = 10).

For each pair, we ran two sessions, each lasting two hours: a control session during which the fish had similar hunger levels and, two days later, an experimental session two hours after satiating the focal fish with 200 mg of bloodworms (corresponding to approximately 30 medium-sized worms). ‘Satiated’ fish consumed 155±3 mg (mean ± SE) out of the 200 mg offered. For each session, a pair was placed in the same experimental tank as used for the temperament assessment, but this time the long partition was made of transparent rather than opaque plastic, so that the fish could see one another. After a five-minute acclimation period, the behaviour of the pair was video-recorded for two hours (without any food in the tank), after which the fish were returned to their compartments in the holding tanks. Fish were fed daily in the compartments with two bloodworms per day.

### Statistical Analysis

**Figure 1:**
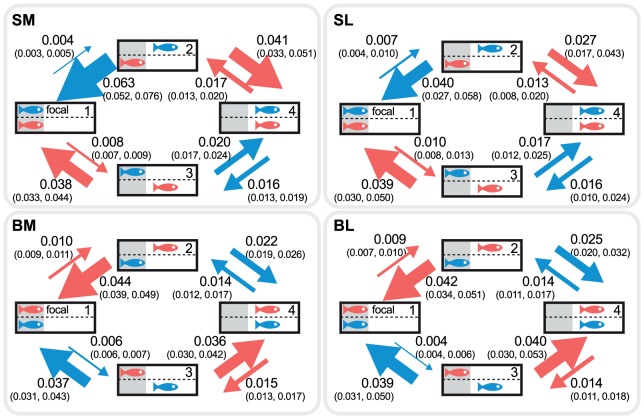
Transition intensities before satiation from the Markov chain model. Transition intensities before satiating focal fish estimated from the Markov chain models (best estimates and 95% CI). SM: focal fish are moderately shyer than their partner before satiation, SL: focal fish greatly shyer than their partner, BM: focal fish moderately bolder than their partner, BL: focal fish greatly bolder than their partner. The area under cover is shaded, while the exposed area is in white. Each state is identified with a number (1–4) at the top-right corner of the tank. Arrow width is proportional to the corresponding value, and arrow colour represents the state transitions of bolder individual (red) and shyer individual (blue) in the pair.

To investigate collective behaviour, we recorded times at which the fish in a pair changed the states based on their positions from the video playback using a custom-made data logger. These states consisted of 1) both fish under cover, 2) focal fish out of cover and partner under cover, 3) partner out and focal fish under cover, and 4) both fish out (figure 1). The time series of transitions between these states were used to fit a continuous-time Markov chain model for each group (package msm 1.0 under R 1.40). Boldness scores of focal and partner fish were included as covariates in the model to improve the overall fit. We estimated transition rates between states and log-linear effects of the focal fish's satiation on both its own transition rates and those of its partner fish (mean and 95% confidence intervals).

From the time series, we also estimated the proportion of time spent out of cover by each fish, as well as the number of distinct trips made out of cover. Trips were classified as attempted initiations (when one fish moved out of cover while the other fish was still under cover), which became joint trips if the other followed, and follows (when a fish followed the other fish out of cover). We compared the proportion of time spent out of cover and the number of distinct trips made before and after satiating the focal fish using paired t-tests (two-tailed).

## Results

### Collective Behaviour before Satiation

**Figure 2:**
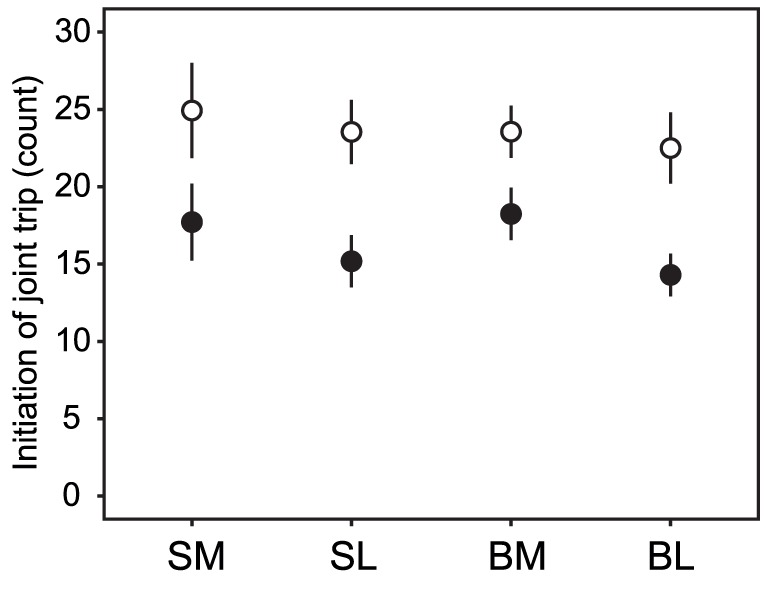
Numbers initiating joint trips before satiation. SM: focal fish are moderately shyer than their partner before satiation, SL: focal fish greatly shyer than their partner, BM: focal fish moderately bolder than their partner, BL: focal fish greatly bolder than their partner. Open circles indicate the bolder individual, and filled circles indicate the shyer individual. Values are means ± SE.

Before satiation, the tendency of pair members to leave cover alone was well predicted by their temperament in isolation. Markov chain models fitted to each group showed greater transition intensities for the bolder member of the pair to leave cover alone (figure 1; *P*≤0.02 for all appropriate transition intensity comparisons; e.g. *q*_12_<*q*_13_ in SM, where *q_ij_* denotes a transition intensity from state *i* to state *j*). On the other hand, no difference between the two members of a pair was seen in their tendency to return to cover when out alone (with the exception of SM, *q*_31_<*q*_21_, *P*<0.001). The higher tendency of the bolder fish to leave cover alone led to the bolder fish initiating the majority of joint trips, with highly significant asymmetries in the number of initiations for pairs with large temperamental differences (paired *t*-tests for SL: *t*_10_ = 6.35, *P*<0.001; BL: *t*_9_ = 3.82, *P* = 0.004; figure 2) and marginal asymmetries for pairs with moderate temperamental differences (paired t-tests for SM: *t*_13_ = 2.17, *P* = 0.050; BM: *t*_24_ = 1.98, *P* = 0.059; figure 2).

### Collective Behaviour after Satiation

**Figure 3:**
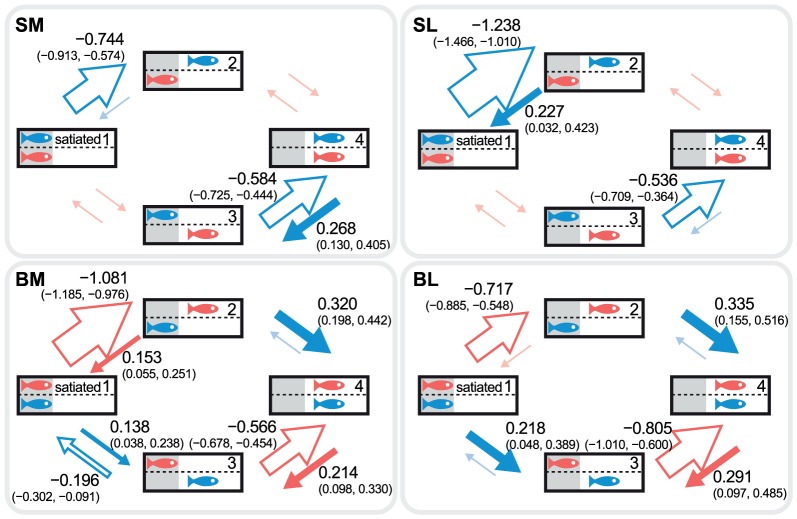
Log-linear effects of satiation from the Markov chain model. Log-linear effects of satiating focal fish estimated from the Markov chain models (best estimates and 95% CI). SM: focal fish are moderately shyer than their partner before satiation, SL: focal fish greatly shyer than their partner, BM: focal fish moderately bolder than their partner, BL: focal fish greatly bolder than their partner. Filled arrows and open arrows in (B) indicate positive and negative effects, respectively.

**Figure 4:**
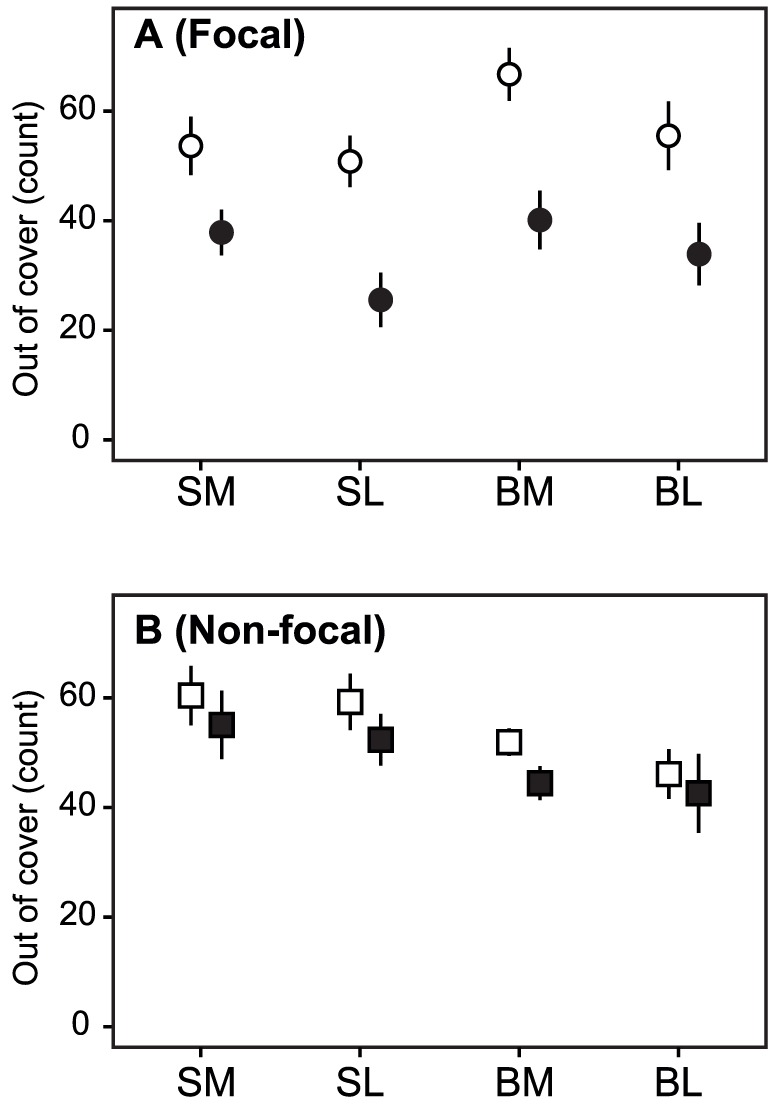
Numbers of trips out of cover (irrespective of whether alone or joined by the partner). (A) Focal fish and (B) non-focal fish. SM: focal fish are moderately shyer than their partner before satiation, SL: focal fish greatly shyer than their partner, BM: focal fish moderately bolder than their partner, BL: focal fish greatly bolder than their partner. Open symbols indicate before satiating focal fish, and filled symbols indicate after satiating focal fish. Values are mean ± SE.

When satiated, individuals strongly decreased their tendency to leave cover, irrespective of the temperament and position of their partner. The effect of satiation was significantly negative for all transitions pertaining to the satiated individual leaving cover (figure 3; *P*<0.05 for all transitions; e.g. *β*_12_<0 and *β*_34_<0 for SM, where *β_ij_* denotes the log-linear effect of satiation). In several instances, satiation also led to a significant increase in the tendency to return to cover (*β*_21_>0 for SL and BM, *P*<0.05; *β*_43_>0 for SM, BM and BL, *P*<0.05), although these effects were generally small compared to the effects on leaving cover (with the exception of BL, *β*_34_ = *β*_43_, *P* = 0.096). The combined effect of these changes was a decrease in time out of cover (paired *t*-tests for SM: *t*_13_ = 3.82, *P* = 0.002; SL: *t*_10_ = 5.25, *P*<0.001; BM: *t*_24_ = 8.08, *P*<0.001; BL: *t*_9_ = 3.62, *P*<0.006) and number of trips out of cover on the part of the focal fish after satiation (paired *t*-tests for SM: *t*_13_ = 3.21, *P* = 0.007; SL: *t*_10_ = 4.96, *P*<0.001; BM: *t*_24_ = 6.16, *P*<0.001; BL: *t*_9_ = 3.02, *P* = 0.014; figure 4).

Non-focal fish, whose partners were satiated, responded differently depending on their relative temperament. Non-focal individuals that (prior to any experimental manipulation) were bolder than their partner did not change their tendency to leave or return to cover when their partner was fed, irrespective of the magnitude of the difference in temperament within the pair [(*β*_13_, *β*_31_, *β*_24_, *β*_42_) = 0 for SM and SL, *P*>0.05]. Indeed, despite the reduction in activity by their satiated partner, bolder non-focal fish did not change their number of trips out of cover (for SM, *t*_13_ = 1.49, *P* = 0.159; for SL, *t*_10_ = 1.29, *P* = 0.227; figure 4). Thus, as expected from a leader who is not overly sensitive to its partner [9], [13], bolder fish maintained their activity levels even when their shyer partner became less active due to satiation.

**Figure 5:**
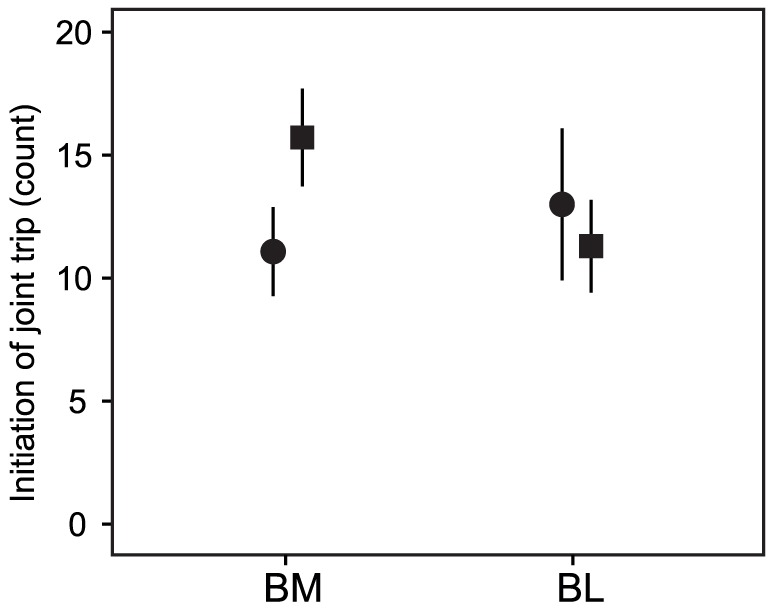
Numbers of initiating joint trips after satiating focal fish. BM: focal fish are moderately bolder than their partner before satiation, BL: focal fish greatly bolder than their partner. Filled circles indicate focal fish (satiated), and filled squares indicate non-focal fish (non-satiated). Values are mean ± SE.

Non-focal individuals that (prior to any experimental manipulation) were shyer than their partner, on the other hand, did change their behaviour in response to the reduced activity of their satiated partner. Irrespective of the magnitude of the difference in temperament within the pair (i.e. BM and BL), shyer fish increased their tendency to leave cover when their partner was fed, both by themselves (*β*_13_>0, *P*<0.05) and in response to their partner being out of cover (*β*_24_>0, *P*<0.05). In the case of shyer fish whose partners were only moderately bolder than them (i.e. BM), we also observed a decreased tendency to return to cover when out alone (*β*_31_<0, *P*<0.05). In other words, shyer fish responded to the decreased activity of their satiated partner by partially taking over the role of initiating trips (i.e. being the first to leave cover), and they were more willing to stay out even in the absence of their partner. For pairs with a large temperamental difference (i.e. BL), where the bolder fish's activity level was still relatively high compared to the shyer fish despite satiation, the change in behaviour by the shyer partner allowed it to maintain its number of trips out of cover at the same level as before the bolder partner was satiated (BL: *t*_9_ = 0.46, *P* = 0.656; figure 4). The increase in activity by the shyer fish in such pairs effectively led to a situation in which the two members of the pair shared leadership, each initiating a similar number of joint trips out of cover (BL: *t*_9_ = 0.59, *P* = 0.568; figure 5). On the other hand, in pairs with moderate temperamental differences, the reduced rate of initiation by the satiated partner meant that the shyer fish made few trips out of cover (BM: *t*_24_ = 3.08, *P* = 0.005; figure 4). In this latter group, satiation actually led to the bolder fish losing its leader role, with the shyer fish tending to initiate a larger number of joint trips (BM: *t*_24_ = 1.90, *P* = 0.070; figure 5).

## Discussion

When pair members had similar hunger levels, temperament was a good predictor of social roles, with bolder individuals taking on a leader role and shyer individuals following. This result is in line with similar observations on fish [21] and birds [22], [31], [32]. However, when individuals differed in their hunger levels, social roles depended on the interaction between hunger and temperament. The bolder member of a pair did not change its behaviour when its shyer partner was satiated, while the shyer member of a pair responded to a decrease in activity level of its bolder partners by taking on a leader role more often.

Bold individuals are known to be less responsive to the actions of their group members, and have a lower tendency to spend time with other group members [9], [33]. Thus, it is not surprising that, when their shyer partner was fed, bolder individuals maintained their role as leader and continued to perform the same number of foraging trips, ignoring their less-motivated companions. More interesting is the reaction of the shyer member of a pair when the former leader (i.e. the bolder member) was satiated. A decrease in initiative by the former leader led to an increase in initiative on the part of the former follower, because the former follower needed to make up for the drop in the number of trips initiated by the former leader. When the temperamental difference between the pair members was small to start with, the former follower (i.e. the shyer member) actually became the new leader. Thus, while temperament is an important predictor of social roles, our results show that such roles are adaptable to circumstances and can be exchanged.

The observation that roles can switch in response to changes in state validates a key qualitative prediction of the model of Rands et al. [17], in which leaders and followers emerged as a result of asymmetries in energy reserves, so that roles could be switched if this asymmetry in state was reversed (i.e. if a hungry leader managed to get much more food than its more satiated follower). Quantitative tests of this model are logistically difficult, as they would require tracking energy reserves and foraging success over long periods of time. However, we were able to confirm that, at least for individuals with similar temperament, asymmetries in foraging success can indeed generate leader and follower roles, and that these can be reversed by manipulating food intake. Such state-driven reversals may be common under natural circumstances because a hungry leader is likely to obtain more food than followers as a result of arriving at a food source earlier [7], [14].

Our results also show that temperament can influence sensitivity to energetic state. Previous work found that hunger affects shoal preferences in shy individuals but has no effect on bold individuals in three-spined sticklebacks [28]; in a similar way, we found that hunger affects the propensity to lead cover in shy individuals but has no effect on bold ones. Most discussions of temperament have focused on consistent individual differences in behaviour, whether in the context of group movement [9], [22], [23], [34] or of other activities [35]–[37]. Our findings suggests that temperament can play a more subtle role in influencing leadership when energetic states fluctuate over time, because it determines the extent to which individuals react to short-term deprivation.

It is noteworthy that bolder members of a pair were less sensitive than shyer members to a partner's behavioural changes. Our previous study also showed that, in the absence of difference in energetic states, bolder individuals are less sensitive to failure in recruiting their partners [13]. This agrees with the theoretical prediction that, when responsiveness is reinforced by positive feedbacks between group members, difference in responsiveness is likely to emerge as a fundamental factor structuring temperamental differences in both social [38] and asocial environments [39]. Furthermore, a model by Wolf et al. [40] showed that social responsiveness can facilitate the emergence of behavioural consistency and vice versa, stabilising the coexistence of socially responsive individuals and behaviourally consistent individuals through a positive social feedback. Our results support their model—shyer individuals were socially aware and behaviourally flexible, while bolder individuals exhibited consistent and unresponsive behaviour. Similarly, in social learning, it is reported that dominant individuals are less influenced by social facilitation than subordinates [41], and that the subordinates copy choices made by the dominant individuals [42]. This difference can be an important factor in maintaining group cohesion [42], and give rise to ‘leaders’ and ‘followers’ in collective learning.

Our results clearly demonstrate that social roles are affected by multiple factors that can act in synergy to determine which individual emerges as leader. This synergetic effect needs to be considered in studies of both coordinated movement [43] and social learning [44]. Most empirical and theoretical studies have concentrated on single predictors, controlling for or ignoring all other factors. Future work will need to integrate different predictors of temperament, and explore how they interact.
